# Supplementary material for: Are the benefits of prosocial spending and buying time moderated by age, gender, or income?
Source: PLoS One. 2022 Jun 9;17(6):e0269636. doi: 10.1371/journal.pone.0269636 (PMC9182255; doi:10.1371/journal.pone.0269636)
Supplement: Attachment — Submitted filename: 04-12-2022 Revision Letter.pdf [file pone.0269636.s001.pdf]

Dear Dr. Galak,

Thank you and the two reviewers for your helpful and constructive feedback on our work. We have uploaded a copy of our revised manuscript. Below, we address each of the comments raised by you and each of the reviewers. We also made some minor changes to sentences that might be unclear or confusing to readers (e.g., confusing wording, typos) throughout the manuscript.

1. **Reviewer 1 was not convinced that "the present research provides the clearest evidence that prosocial spending and buying time is robustly related to happiness across demographic groups," citing past research that have involved more diverse demographics (e.g., Aknin et al., 2013). Along similar lines, Reviewer 2 asked us to clarify what we mean by "diverse."**

We thank the reviewers for pointing this out. In response, we have improved the precision of our claims. Specifically, we now clarify that "by drawing on a large and *economically* diverse sample of Americans, the present research provides some of the clearest evidence to date that prosocial spending and buying time are robustly related to happiness *within the United States*" (p. 21). We have also made changes throughout our manuscript to ensure that we are not overstating the diversity of our sample.

2. **Reviewer 1 wanted to understand why we combined positive mood and life satisfaction to create an overall measure of subjective well-being. They also asked for more precision about what we meant by "emotional benefits" throughout the manuscript.**

In his seminal work, Ed Diener (1984) defined subjective well-being as a combination of two distinct but related components: a person's (1) affective and (2) cognitive evaluation of their own life. In line with this widely accepted definition, the company assessed subjective well-being by combining our measures of positive mood and life satisfaction. In our revised manuscript, we explain this approach on p. 10.

Subjective well-being and happiness are used interchangeably in well-being research. Thus, we have replaced all references to "emotional benefits" with "SWB" or "happiness benefits" throughout our manuscript (e.g., p. 16).

3. **Reviewer 1 pointed out that asking participants whether buying something for others versus themselves brings them more happiness measures a lay theory rather than spending preferences.**

In line with Reviewer 1's comments, we changed the name of our measure to "lay theories about spending money on others" (p. 12). We also made changes throughout our manuscript to be consistent with this new terminology.

4. **Reviewer 1 was curious whether the effect of income on SWB was stronger than the effect of making time-saving purchases. To that end, they asked to report the correlation between time-saving purchases and income.**

As described on p. 14, the relationship between buying time and subjective well-being remains significant even after controlling for personal income. Thus, personal income cannot fully explain the happiness benefits of buying time. Still, in light of Reviewer 1's feedback, we added a table that reports the correlation between all of our key variables in p. 9.

5. **Reviewer 1 asked us to clarify what was predicted versus exploratory, suggesting that we use more precise language to describe our results.**

We did not pre-register any of our analyses and thus, our results should be interpreted as exploratory. In addition to highlighting this point in our general discussion (p. 22), we have removed any statements suggesting that we made a prediction in advance (e.g., "but in an unexpected direction" as highlighted by the reviewer).

6. **Reviewer 1 felt the first sentence of our abstract was a bit out of place given the content of our manuscript.**

Following Reviewer 1's suggestion, we have modified the first sentence of our abstract.

7. **Reviewer 2 shared some additional context around the literature on buying experiences that is currently missing from our introduction.**

We thank Reviewer 2 for providing this additional context. In our revised manuscript, we added this information to our introduction about the literature on buying experiences (p. 2). We also clarify that our point about "not knowing whether these spending strategies should be widely recommended" is about prosocial spending and buying time (rather than buying experiences which has been studied in greater detail).

8. **Reviewer 2 suggested that we use a more informative title that provides some answers in the form of a declarative statement (vs. asking a question).**

Following Reviewer 2's suggestion, we considered an alternative title for our manuscript: "Prosocial spending and buying time is related to subjective well-being across age, gender and income groups in the United States." However, we believe this title oversimplifies our results and that no declarative statement would sufficiently capture the nuances in our data. Thus, we stuck with our current title. However, we are happy to use an alternative title if you prefer it.

Please let us know if you feel that we have misunderstood or inadequately addressed any of the comments above. Thank you again for your feedback on our manuscript!

Sincerely,

Iris Lok
University of British Columbia
Iris.lok@psych.ubc.ca

Elizabeth Dunn
University of British Columbia
profdunn@gmail.com
